# Retracted: To Explore the Effects of Acupuncture and Medical Treatment at Different Times on the Gastrointestinal Reaction and White Blood Cell Count of Patients with Lung Cancer Chemotherapy

**DOI:** 10.1155/2023/9892836

**Published:** 2023-03-09

**Authors:** Applied Bionics and Biomechanics

*Applied Bionics and Biomechanics* has retracted the article titled “To Explore the Effects of Acupuncture and Medical Treatment at Different Times on the Gastrointestinal Reaction and White Blood Cell Count of Patients with Lung Cancer Chemotherapy” [1] due to concerns that the peer review process has been compromised.

Following an investigation conducted by the Hindawi Research Integrity team [2], significant concerns were identified with the peer reviewers assigned to this article; the investigation has concluded that the peer review process was compromised. We therefore can no longer trust the peer review process and the article is being retracted with the agreement of the Chief Editor.

The authors do not agree to the retraction.
